# Chitosan-Stabilized Noble Metal Nanoparticles: Study of their Shape Evolution and Post-Functionalization Properties

**DOI:** 10.3390/nano10020224

**Published:** 2020-01-28

**Authors:** Massimo Ottonelli, Stefania Zappia, Anna Demartini, Marina Alloisio

**Affiliations:** 1 Dipartimento di Chimica e Chimica Industriale, Università di Genova, Via Dodecaneso 31, 16146 Genova, Italy; massimo.ottonelli@unige.it (M.O.); stefania.zappia@ismac.cnr.it (S.Z.); adema.chimica@gmail.com (A.D.); 2 Istituto di Scienze e Tecnologie Chimiche “Giulio Natta” (SCITEC), Consiglio Nazionale delle Ricerche (CNR), via Alfonso Corti 12, 20133 Milano, Italy

**Keywords:** anisotropic noble metal nanoparticles, plasmonic properties, chitosan, galvanic replacement reactions, spontaneous reshaping, diacetylenes

## Abstract

Noble metal anisotropic nanostructures have achieved a growing interest in both academic and industrial domains mostly because of their shape-dependent plasmonic properties in the near-infrared region. In this paper, gold and gold-silver anisotropic nanostructures were synthesized in very high shape-yields through a wet, seed-mediated approach based on the use of nearly spherical silver nanoparticles as seeds and chitosan as stabilizing agent. Two chitosans of different origin and molecular properties were selected for the synthetic pathway, leading to the formation of variously sized and shaped end products. In detail, quite homogeneous nanoplatelets of about 25-nm size and 7-nm thickness or nearly spherical, highly porous nanocages of about 50-nm size were obtained, depending on the type of polysaccharide employed. The shape transition towards anisotropic morphologies occurred through a slow, spontaneous process, in which the chitosan nature seemed to play a key role. As expected, both nanoplatelets and nanocages exhibit shape-dependent plasmonic features and surface properties tunable for a variety of application fields. To prove this point, the nanostructures were successfully post-functionalized with poly(10,12-pentacosadiynoic acid) (PCDA), a carboxylic-endowed diacetylene able to anchor on noble metal substrates, to obtain versatile, chromic platforms suitable for sensing and spectroscopic purposes.

## 1. Introduction

Anisotropic noble metal nanostructures (NSs) represent a particularly fascinating class of nanomaterials because of their size- and shape-dependent physical and chemical properties, which make them ideal candidates for the development of new technologies [1]. In particular, higher aspect ratio NSs are of utmost interest for applications involving manipulation of light due to their multiple surface plasmon resonances (SPRs), the position of which can be fine-tuned within the visible and near-infrared region by properly controlling the nanoparticle shape [2]. When optical absorption is achieved in the NIR interval, that is the wavelength window where blood and tissues are relatively transparent to the radiation, these plasmonic nanostructures can be profitably used in diagnostic and therapeutic medical applications, as well documented in the literature [3]. Moreover, owing to the strong, localized electromagnetic fields generated in their proximity, NSs were successfully exploited as surface-enhanced Raman spectroscopy (SERS) substrates [4] or highly performing platforms for chemical and biological detection [5] and nonlinear optical (NLO) applications [6]. Finally, the anisotropy of NSs was proved to help their self-assembly into ordered 2D and 3D lattices with spatially dependent properties at the macroscale level, which still constitutes a challenging issue in nanomaterials research [7,8].

In view of their promising use in such various fields, a strict control of the size and shape of anisotropic noble metal nanoparticles is required. Among the available schemes of synthesis following a bottom-up approach, the so-called seeded-mediated growth method in aqueous solution [9] is particularly popular because of its versatility in producing nanoparticles of different geometry in high shape yields. The method is based on the selective reduction of a gold precursor (growth solution) on premade seeds, which act as both catalysts and nucleation points for the growth of the gold nanostructures. If the metal reduction is carried out in a controlled way involving symmetry-breaking events, the particle growth along specific directions is favored and products with anisotropic features are formed. Initially proposed for the quantitative synthesis of gold nanorods with a tailored aspect ratio [10,11], this route was then successfully exploited to produce differently shaped particles such as nanostars [12], nanotriangles [7,8], and nanoplatelets [13]. In most seeded-mediated grown protocols, the presence of a capping agent acting as a stabilizer is required. The nature of the capping agent is crucial to drive particle growth in the nanometer regime, most probably through the selective adsorption of stabilizer to specific facets of the metal core. Consequently, various types of capping agents were tested, such as surfactants, ligands, polymers, or dendrimers [14] with alternate results in terms of shape control. Recently, we succeeded in obtaining triangular gold nanoplates in shape yields up to 65% by the spontaneous reshaping of nearly spherical seeds induced by a cationic diacetylene employed as unconventional surfactant [15]. Alternatively, the shape control of noble metal nanoparticles can be achieved using templates of defined geometry. Since the templates act as spatial constraints during the growth process, the final shape of the nanoparticles is determined by that of the pristine substrates [16]. Among the template-assisted strategies to synthesize noble metal NSs, the galvanic replacement reactions, in which the difference in the standard electrode potentials of two elements is exploited to lead to the deposition of the more noble component and the dissolution of the less noble one, were widely used to make gold-based hollow nanostructures with different morphologies including nanocages [17], nanocubes [18], nanoboxes [19], single-walled [20] or multiple-walled [21] nanotubes. In the past, we took advantage of this approach to prepare stable nanocages in Au-Ag alloy with extended plasmonic features to the NIR region by titrating a hydrosol sample of nearly spherical, chitosan-stabilized silver nanoparticles (CS@AgNPs) with an aqueous solution containing Au^3+^ ions [22]. The choice of chitosan (CS) as the stabilizing agent was dictated by several reasons. Chitosan is a low cost, renewable polysaccharide deriving from the partial deacetylation of chitin, which is the second most abundant natural polymer in the world. The polycationic nature gives to CS solubility in dilute acidic solutions, ease of chemical modification, and hosting capabilities for entrapping noble metal nanoparticles thanks to the specific interactions established within the amino substituents of the polymer and the metal surfaces [23]. Because of its low toxicity, tailored biodegradability, and high biocompatibility, chitosan is widely used in various application domains [24]. In particular, its antibacterial, antifungal, mucoadhesive, wound healing, and even anticancer activities are well recognized and deeply exploited in the pharmaceutical and biomedical areas [25]. For these reasons, the European Commission-DG RTD, operating in the framework for mapping innovative products deriving from biomass components, recently inserted third-generation chitosan in the top 20s emerging bio-based products (BBS) to be used in industrial applications for the development of highly demanding systems [26].

Starting on these bases and with the aim of combining the intrinsic properties of CS with those of noble metal NSs to produce novel platforms with implemented biocompatibility and plasmonic features, we investigated the capability of chitosan to act as shape-directing agent in the fabrication of gold-based nanostructures through a standard galvanic replacement route involving nearly spherical silver nanoparticles as seeds. Two commercial chitosans of different origin, molecular mass and acetylation degree (AD), σCS and HCS, were selected to study the influence exerted by the molecular properties of the polysaccharide on the synthetic process previously set up by us to obtain nanocages in Au–Ag alloy [22]. In this way, nanoplatelets of varied geometry or spherical-like nanocages were obtained depending on the chitosan employed. In both cases, the end products were the results of a shape transition that spontaneously occurred in water at room temperature and required about a year to be accomplished.

Morphological and spectroscopic characterization of the samples, carried out through several techniques, revealed that the nanostructures exhibited plasmonic features closely related to their anisotropic shape and then suitable for a large variety of applications. Moreover, the spectroscopic properties of these nanostructures are expected to be further implemented by post-synthetic functionalization with accessory chromophores or fluorophores. To prove this point, the chitosan-protected NSs were successfully coated with 10,12-pentacosadiynoic acid (PCDA), a carboxyl-endowed diacetylene able to firmly anchor on noble metal substrates to form core-shell nanoassemblies with outstanding optical properties exploitable for sensing and NLO purposes [27,28,29].

## 2. Materials and Methods

Reagents, solvents and glassware. Chemicals and spectroscopic grade solvents were commercial products mostly used as received. Highly viscous chitosan from crab shells (σCS, Sigma-Aldrich, St. Louis, MO, USA) and high molecular mass chitosan (HCS, Fluka) were purified prior to use through a three-step procedure consisting in (1) precipitation in a coagulation bath of EtOH:NH_4_OH (28% *v*/*v*):H_2_O (7:3:1 *v*/*v*), (2) cooling in liquid nitrogen and (3) lyophilization. Once purified, the chitosans were characterized by means of viscometry and FTIR spectroscopy in order to evaluate the mean molecular mass (${\bar{M}}_{\nu }$) and the acetylation degree (AD). As reported elsewhere [30], the values of ${\bar{M}}_{\nu }$ = 9.6 × 10^5^ g/mol and AD = 33.8% were evaluated for σCS and the values of ${\bar{M}}_{\nu }$ = 3.4 10^6^ g/mol and AD = 22.8% were evaluated for HCS. The diacetylene monomer 10,12-pentacosadiynoic acid (PCDA, Sigma-Aldrich, St. Louis, MO, USA) was purified prior to use from spontaneously formed blue polymer by means of dissolution in ethanol followed by filtration with 0.20-µm PTFE syringe filter. 

Aqueous solutions were prepared with ultra-high-purity Milli-Q water distilled twice prior to use. 

Before the synthesis of the nanostructures, the glassware was thoroughly cleaned with freshly “piranha” solution prepared by mixing cooled hydrogen peroxide (30% *v*/*v*) and concentrated sulfuric acid in the ratio of 1:2 *v*/*v* and then rinsed with bi-distilled water for immediate use.

Instrumentation. Electronic absorption spectra of colloidal suspensions were acquired at room temperature through a Perkin-Elmer Lambda 9 (Bodenseewerk Perkin-Elmer & Co GmbH, Uberlingen, DEU) and a Shimadzu UV-1800 (Shimadzu USA Manufacturing, Inc., Canby, OR, USA) spectrophotometer with fused silica cuvettes of different path length.

Micro Raman spectra of hydrosols were obtained by a Renishaw RM2000 single grating spectrograph, (Renishaw Ltd., Gloucestershire, GBR) equipped with a Leica Microscope DMLM (20× confocal lens, 1–9 accumulations) (Leica Microsystems Digital Imaging, Cambridge, GBR). Irradiation of the samples was accomplished by means of a diode laser source emitting at 623 nm, the power density of which was reduced from 1 to 25% by properly defocusing the 3-mW laser beam. 

FT-IR spectra were acquired on dried or powder samples by means of a Bruker Vertex 70 instrument (Bruker Optik, GmbH, Ettlingen, DEU), operating in an attenuated total reflectance (ATR) mode.

Analysis through atomic absorption spectrometry was carried out employing a Spectra AA 55B Varian spectrometer (Varian Australia Pty Ltd., Mulgrave Victoria, AUS).

Field emission scanning electron microscopy (FESEM) images of the nanostructures were recorded using a ZEISS SUPRA 40 VP microscope (Carl Zeiss NST, GmbH, Oberkochen, DEU) operating at 20 keV in both direct (InLens mode) and back (QBSD mode) configuration. Before the morphological characterization, the samples were sputter-coated with carbon using a Polaron E5100 sputter coater (Polaron Co., Watford, GBR) to obtain good conductivity. A statistical analysis of the nanostructure’s size and shape was carried out on at least 100 measurements, taken from FESEM images with the open-source software Image J, by employing the Kolmogorov-Smirnov [31] and the median absolute deviation (MAD) [32] tests, as detailed in a previous work [33].

The rheological characterization of hydrosols was performed using a rotational rheometer Physica MCR 301 (Anton Paar, GmbH, Graz, AUT). A Peltier heating system coupled with a solvent trap kit was employed to set the temperature at 25.0 ± 0.2 °C and prevent the solvent evaporation. A cone-plate geometry (CP50) with a 50-nm diameter, a 1° angle, and a 99-μm truncation was used. Photopolymerization of the samples was carried out in a Rayonet photochemical chamber reactor, Model RPR-200 (Sprindler & Hoyer, GmbH, Göttingen, DEU) operating at 254 nm and 35 W, by keeping the hydrosol-containing cuvettes at a 10-cm distance from the UV lamps.

Synthesis of chitosan-stabilized noble metal nanostructures. Noble metal nanostructures stabilized with σCS (σCS@NSs) or HCS (HCS@NSs) were prepared through a wet seed-mediated grown approach following a synthetic procedure already employed by us to produce chitosan-protected nanocages in Ag–Au alloy [22]. Previously, hydrosols of σCS-protected or HCS-protected silver nanoparticles (σCS@AgNPs and HCS@AgNPs, respectively) of nearly-spherical shape and nominal concentration of 5 mmol/L in terms of Ag content were obtained in water at room temperature by reducing AgNO_3_ (0.8 mL, 80 mmol/L) with a freshly prepared aqueous solution of NaBH_4_ (0.8 mL, 80 mmol/L) in the presence of chitosan dissolved in 1% *v*/*v* acetic acid solution (5 mL, 62 mmol/L in terms of repeating units). The solutions, which immediately became opalescent yellow suspensions, were maintained under stirring for approximately 1 h to ensure full reaction. Subsequently, 12 mL of the as-prepared silver hydrosols were dispersed in 100 mL of bi-distilled water, refluxed at the boiling point for 1 h and then added dropwise of a proper aliquot of HAuCl_4_ aqueous solution (0.4 mL, 30 mmol/L) under magnetic stirring. The suspensions, which turned out first colorless and then deep dark, were heated for another 20 min, cooled to room temperature and kept under vigorous stirring overnight. The crude products of a nominal concentration of about 0.5 mmol/L and 0.1 mmol/L in terms of Ag and Au contents were centrifuged at 10k rpm for 10 min, re-dispersed in water and then stored at room temperature. Coloration changes were observed in the hydrosols over time, which can be taken as an insight of morphology transitions spontaneously occurring in the nanostructures. After a year of time, the σCS@NSs-containing sample assumed a stable grey-blue tint, whereas the HCS@NSs-containing one appeared purple-brown colored (Figure S1a in Supplementary Materials). Color changes and stability of the samples were monitored by means of UV-vis-NIR and FTIR-ATR spectroscopies (Figure S1b,c in Supplementary Materials).

Synthesis and polymerization of PCDA-coated noble metal nanostructures. Noble metal nanostructures coated with PCDA molecules (PCDA@NSs) were obtained by chemisorption of the diacetylene on as-prepared chitosan-protected precursors by means of a ligand-exchange reaction (LER) protocol set up by us to produce core-shell nanohybrids in water [27,28,29,34]. The procedure was applied to both fresh and aged σCS@NS as well as to aged HCS@NSs under the same experimental conditions adopted in the past in order to compare the NSs behavior with that of previously studied nanoparticles. In detail, 5 mL of hydrosol containing σCS@NSs or HCS@NSs were diluted with 5 mL of bi-distilled water and added 30 μL of PCDA dissolved in ethanol (9 mmol/L). The mixtures were maintained seven days in the dark at room temperature under constant stirring to allow the reaction to complete. Afterwards, the samples were placed in a fused silica cuvette and subjected to UV light for 1 min in a Rayonet chamber to induce the photopolymerization of the diacetylene shell and then stored in the dark at room temperature without further purification.

For the sake of comprehension, the names given to the samples are explained in Table S2 in Supplementary Materials.

## 3. Results

### 3.1. Characterization of Chitosan-Stabilized Nanostructures

**Figure 1:**
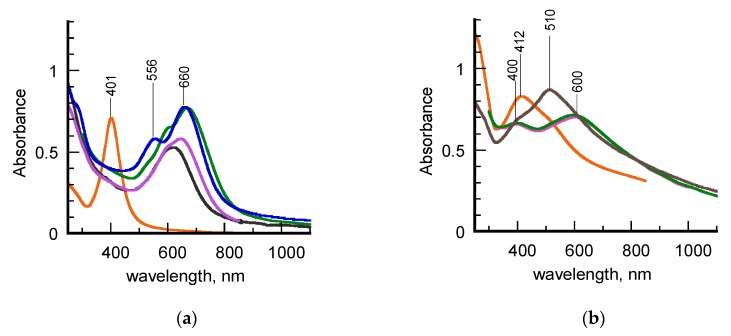
(**a**) UV–vis–_NIR spectra of hydrosols of σCS@AgNPs (orange line) and σCS@NSs at different aging times: fresh (black line), 14 days (purple line), 75 days (green line), 1 year (blue line). (**b**) UV-vis–NIR spectra of hydrosols of HCS@AgNPs (orange line) and HCS@NSs at different aging times: 35 days (purple line), 60 days (green line), 1 year (brown line).

The UV-vis-NIR spectra recorded for hydrosols of σCS@NSs and HCS@NSs during a year of time are reported in Figure 1. The spectra of the corresponding chitosan-stabilized AgNPs, used as seeds in the synthetic protocol, are added to facilitate the comparison. It is evident that the two samples exhibit a different behavior with aging. In detail, fresh σCS@NSs show a single, nearly symmetric surface plasmon resonance (SPR), characterized by a quite flat maximum centered at 630 nm (Figure 1a, black line). No significant evidence of the plasmon band of the silver precursors around 400 nm are found even in the first steps of the nanostructure formation. Significant changes are observed in the spectral profile over time, in that the SPR band becomes progressively more asymmetrical and red-shifted (Figure 1a, purple and green lines). After a year of aging, the plasmon resonance is split in two distinct components, positioned around 570 and 660 nm, respectively (Figure 1a, blue line). Based on the current literature, the SPR splitting can be attributed to the presence of two populations of isotropic nanoparticles with different sizes or, alternatively, to the formation of a single population of anisotropic nanostructures. In the latter case, the higher energy band around 570 nm corresponds to the transversal component of the plasmon resonance, whereas the lower energy band at 660 nm is assigned to the longitudinal component, which is known to be strictly associated to the onset of anisotropy in noble metal nanoparticles. Anyway, the spectra evolution over time clearly indicates that a spontaneous shape transition of σCS@NSs occurred. The contemporary color change of the σCS@NSs-containing hydrosol from the initial dark color to the final brilliant grey-blue one allowed to detect this shape transition also with the naked eye (Figure S1 in Supplementary Materials).

Different trends and features are observed in the spectra of Figure 1b. First of all, one month-aged HCS@NSs exhibit a spectral profile characterized by the presence of two SPR bands, positioned around 400 and 600 nm, respectively (Figure 1b, black line). According to our previous work [21], these spectral features are consistent with the presence of nanocages in Ag-Au alloy, characterized by truncated corners, hollow interiors, and quite homogeneous walls. Taking into account that HCS@NSs were prepared under the same experimental conditions adopted in the past, this result confirms the good reproducibility of our synthetic method. The spectral profile looks unaltered for about three months (Figure 1b, green line) to indicate that HCS@NSs hydrosol are stable during this time interval, as already experimented [22]. However, significant changes in the spectrum are observed at a year of aging (Figure 1b, brown line), in that the maximum of the low energy SPR band is blue-shifted to 510 nm, a wavelength value typical of solid, gold nanoparticles with isotropic geometry. This finding is consistent with a partial collapse of the nanocages into fragments [16]. Once again, the color transition of the hydrosol from blue-brown to red-brown visually revealed the shape modifications in HSC@NSs (Figure S1 in Supplementary Materials).

Since σSC@NSs and HSC@NSs were obtained under the same experimental conditions, the differences found in their plasmonic features can only be due to the different types of chitosan used in the preparation pathway. The effect of the polysaccharide characteristics, in terms of molecular mass and acetylation degree, is already evident in the spectra of the silver seeds (Figure 1a,b, orange lines). Indeed, whereas the σCS@AgNPs exhibit a highly symmetric SPR lineshape, typical of quite monodisperse isotropic nanostructures, the more irregular spectral profile found for HCS@AgNPs hydrosol suggests that these seeds are characterized by increased heterogeneity in terms of size or shape.

**Figure 2:**
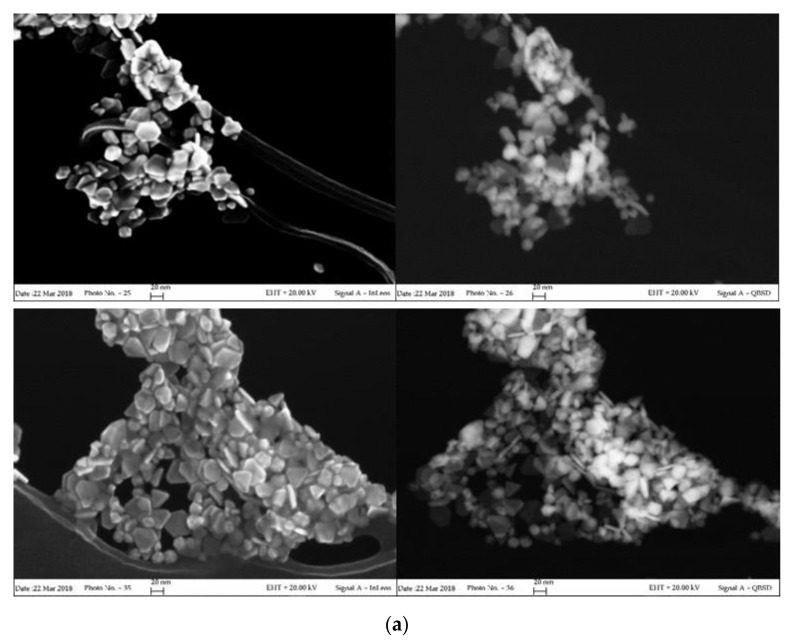

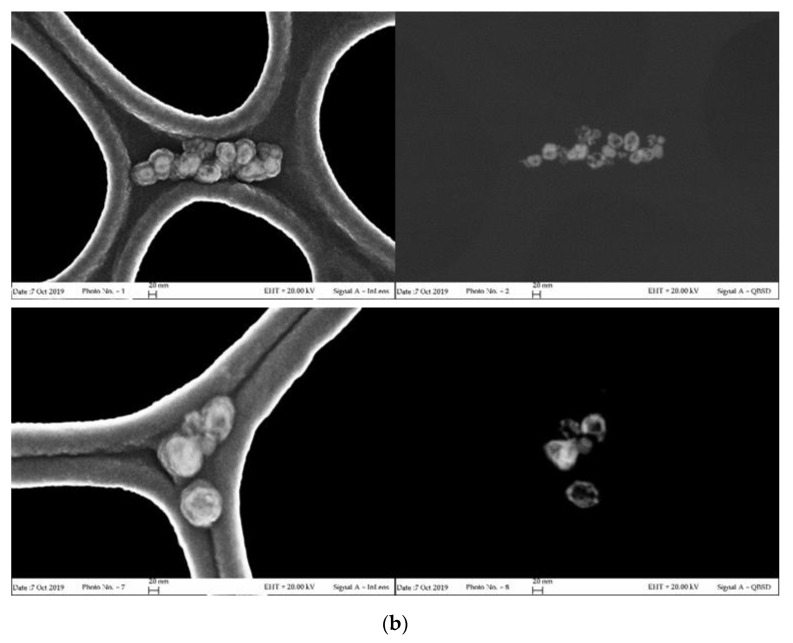
Field emission scanning electron microscopy (FESEM) images of aged σCS@NSs (**a**) and HCS@NSs (**b**) acquired in direct (InLens mode, left) and back-scattered (QBSD mode, right) configurations.

In order to highlight the correlation between spectroscopic properties and morphology, a FESEM characterization of the aged samples was carried out. High magnification images, acquired in both direct and back-scattered configuration to better discriminate the metal noble components, are shown in Figure 2. The results of the size and shape analysis carried out on more than 100 nanoparticles are listed in Table 1. Corresponding distribution histograms are instead reported in Figure S2 of Supplementary Materials.

As clearly evidenced in the photographs of Figure 2a, σCS@NSs are composed by flat, solid structures (platelets) in very high shape yield (around 90%). The platelets, which exhibit both irregularly truncated and triangular geometries in almost equivalent percentages, have average dimensions of about 24 nm and thickness around 7 nm with limited size dispersion. The anisotropic morphology of σCS@NSs, corresponding to an average aspect ratio of 3.3, is consistent with the bidental profile of the corresponding plasmon band, as confirmed by the current literature [7,35]. Nearly spherical particles with increased size (around 55 nm) are instead found for HCS@NSs, as shown in the photographs of Figure 2b. The porous structure revealed by the back-scattered images indicates that the sample is actually composed of nanocages, confirming the results previously published [22]. Evidence of metal core fragmentation are also present, in agreement with the plasmonic features of the corresponding spectrum.

Interesting information on the nanostructures arose from the punctual elemental analysis carried out by means of Energy Dispersive X-Ray Spectroscopy (EDS) supplied by the FESEM technique. The spectra shown in Figure S3 of Supplementary Materials indicate different compositions of the samples, in that silver and gold were found in HCS@NSs, whereas only gold atoms were detected in σCS@NSs. This result, once again consistent with the spectrum of aged HCS-protected nanoparticles, was further confirmed by characterization through atomic absorption spectroscopy (Table S1 in Supplementary Materials). Also in this case, silver was detected only for HCS@NSs in small amounts and almost inverted Au:Ag ratio with respect to the nominal contents of the two metals in the reaction medium.

**Figure 3:**
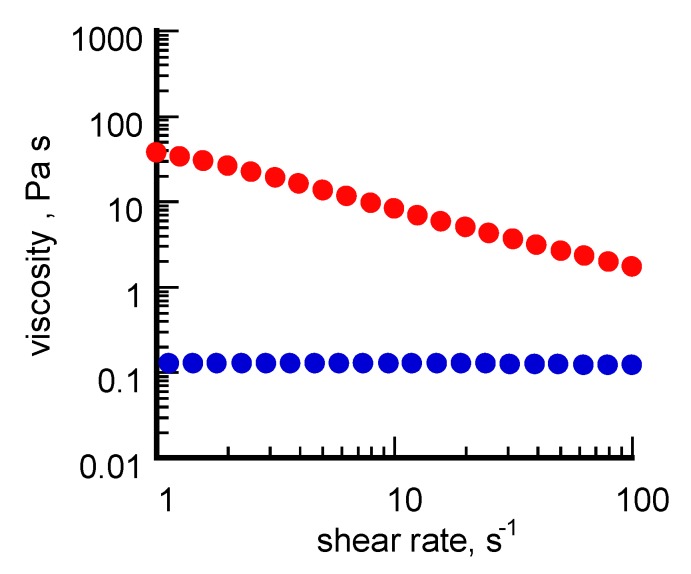
Curves of viscosity (η) obtained from σCS (red line) and HCS (blue line) in water at the 2% g/v concentration in 2% *v*/*v* acetic solution.

Taken together, these data suggest that an aging-induced shape evolution occurred differently for σCS@NSs and HCS@NSs, most likely induced by the chitosan type used in the synthetic pathway as stabilizing/capping agent. Indeed, whereas the nanocages could be produced according to the reaction mechanism proposed by Sun and co-workers [17], a more complex reshaping process is supposed to be involved in the formation of the solid platelets. It is logical to conjecture that the shape evolution route followed by the nanostructures was somehow influenced by the molecular properties of the type of chitosan involved in the synthetic pathway. By taking into account the well-known chitosan ability to gel in aqueous suspensions and how these gelling capabilities are directly influenced by the polysaccharide composition [36], we think that the viscosity of the chitosan solutions, which in turn is ruled by the percentage of free amino groups on the polysaccharide backbone, could be a crucial factor in conditioning the transition of isotropic seeds towards different geometries. To prove this point, a rheological characterization of σCS and HCS in water was undertaken. The curves of viscosity (η) obtained from this study are reported in Figure 3.

The two chitosans exhibit quite different viscoelastic properties. HCS, characterized by higher molecular mass and lower acetylation degree, shows a Newtonian behavior associated with a low viscosity value, at least within the interval of shear rates explored. σCS, instead, shows definitely higher η values in agreement with the manufacturer’s declaration and a pseudoplastic behavior typical of “soft” gels. By taking into account the key role played by the environmental medium in conditioning the final shape of gold nanostructures during seeded-mediated grown methods [37], it is likely that within the gel network formed by σCS in water, the complete dissolution of the silver atoms followed by the spontaneous reshaping of the gold cores towards flat structures is favored. Hereafter, further studies will be addressed to better understand the mechanistic aspects involved in the platelet formation, also by means of a computational approach.

In virtue of their shape-dependent plasmonic properties, the nanostructures can find profitable use in various application purposes, such as performing surface-enhanced Raman scattering (SERS) substrates or colorimetric sensing platforms, also through tailored post-synthetic reactions as demonstrated in the next chapter.

### 3.2. Characterization of PCDA-Coated Nanostructures

PCDA molecules were self-assembled on σCS@NSs and HCS@NSs following a wet incubation procedure detailed in the Materials and Methods section. In this way, diacetylene-coated nanostructures (σ-PCDA@NSs and H-PCDA@NSs, respectively) were prepared and then subjected to UV light for 1 min to produce polymerized nanohybrids (σ-pPCDA@NSs and H-pPCDA@NSs, respectively). In order to investigate the correlation between shape and chemical properties of noble metal nanostructures, the protocol was applied on both fresh and aged σCS@NSs and on aged HCS@NSs.

**Figure 4:**
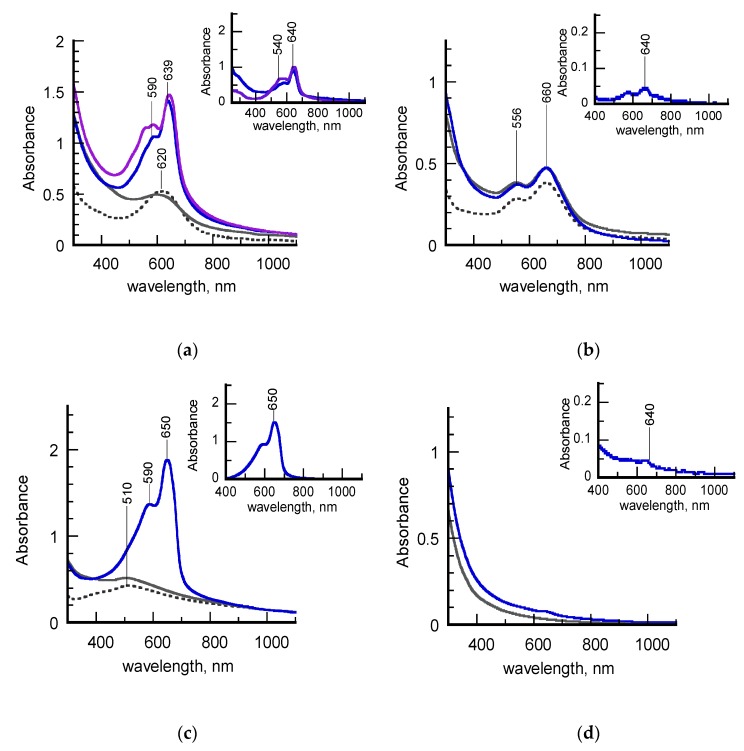
(**a**) UV-vis-NIR spectra of a freshly-synthesized hydrosol of σCS@NSs (dashed grey line), σ-PCDA@NSs (grey line), σ-pPCDA(blue line) and one year aged σ-pPCDA (purple line); the inset highlights the spectral contribution of the poly (PCDA) component; (**b**) UV-vis-NIR spectra of an aged hydrosol of σCS@NSs (dashed grey line), σ-PCDA@NSs (grey line), σ-pPCDA(blue line); the inset highlights the spectral contribution of the poly (PCDA) component; (**c**): UV-vis_NIR spectra of an aged hydrosol of HCS@NSs (dashed grey line,) H-PCDA@NSs (grey line), H-pPCDA@NSs (blue line); the inset highlights the spectral contribution of the poly (PCDA) component; (**d**) UV-vis-NIR spectra of PCDA dispersed in an aqueous solution before (grey line) and after (blue line) photopolymerization.

Figure 4a–c shows the UV-vis-NIR spectra of the investigated hydrosols before and after UV treatment together with those of the pristine σCS@NSs and HCS@NSs. With the aim of facilitating the data discussion, the poly (PCDA) contributions to the overall profiles, obtained by subtracting point-to-point the spectra of the unpolymerized samples to those of the polymerized ones, are highlighted in the insets. Finally, the spectra acquired for the reference sample, corresponding to an aqueous suspension of free PCDA at the same concentration, are also reported (Figure 4d).

It is evident that the samples exhibit different behaviors upon photoirradiation. Before being subjected to UV light, all the PCDA-coated nanostructures (grey lines, Figure 4a–c) retain the plasmonic features of the chitosan-protected precursors (dashed grey lines) to indicate that the morphology of the metal cores was not altered by the anchoring of the diacetylene molecules. On the contrary, significant changes are observed in the spectra of the photopolymerized hydrosols (blue lines). In detail, the spectral lineshapes of both freshly-synthesized σ-PCDA@NSs and aged H-PCDA@NSs are dominated by an intense, sharp peak centered at 640 or 650 nm, followed by a sideband positioned around 590 nm (blue lines, Figure 4a,c). These spectroscopic features can be assigned to the exciton band and its vibronic replica, respectively, of poly (PCDA) in the highly-conjugated blue form. The plasmon contributions to the spectra are no longer detectable, most probably because they are covered by the intensity of the polymer absorptions. With the aim of acquiring information about the stability of polymerized samples, the spectrum of σ-pPCDA@NSs was also recorded a year after preparation (purple line, Figure 4a). The new spectrum almost overlaps the old one, except for the appearance of a satellite band around 540 nm, typical of polydiacetylenes in the less conjugated red form. The contemporary presence of both blue and red excitons in the spectral profile suggests that a partial blue-to-red phase transition spontaneously occurred in the polymeric shell over time, as experimented in the past [28].

Unlike the as-discussed counterparts, aged σ-pPCDA@NSs do not show remarkable changes in the UV-vis-NIR spectrum after irradiation (blue line, Figure 1b). Indeed, the spectral profile reported in the corresponding inset reveals the presence of photogenerated blue poly (PCDA) in a small amount. Overall, the polymerization features of aged σ-pPCDA@NSs resemble those of the reference sample depicted in Figure 4d, in which PCDA molecules are supposed to polymerize in the form of spontaneously self-assembled micelles.

As demonstrated in previous works [27,28,33], semi-quantitative information on the PCDA polymerization can also be achieved from the spectra of Figure 3, by means of blue percentage (PB) and polymerization degree (PD) parameters. The PB parameter, defined as the ratio of the absorbance at the blue exciton wavelength with respect to the sum of absorbances at the blue and red exciton wavelengths, indicates the percentage of blue form in the polydiacetylene skeleton. The PD parameter, calculated by dividing the area of the deconvoluted poly (PCDA) spectrum by the monomer concentration, is effective to correlate the monomer-to-polymer conversion with the intensity of the polydiacetylene absorptions. The PB and PD values for the samples examined in this work are listed in Table 2. The data obtained for photopolymerized PCDA-coated silver nanoparticles (pPCDA@AgNPs) in a previous article [27] are added for the sake of comparison.

The highest values of PB and PD found for aged H-pPCDA@NSs suggest that PCDA self-assembly and subsequent photopolymerization in the blue form are optimized on these nanostructures. This assumption is confirmed by the fact that the same values were calculated for pPCDA@AgNPs, which were individuated as highly performing platforms for the dense stratification of the diacetylene in bilayered shells [29]. The lower values of both parameters related to freshly-synthesized σ-pPCDA@NSs indicate that the photogeneration of blue poly (PCDA), although qualitatively considerable, is more difficult on these substrates. In particular, the PB indicator is effective in evidencing the presence of the red phase not easily detectable in the corresponding spectral lineshape (blue lines, Figure 4a). A dramatic PD drop of about one order of magnitude was instead calculated for aged σ-pPCDA@NSs, associated with a less significant PB decrease. The PD value is even lower than that found for PCDA micelles in water, in which however red and blue poly (PCDA)s are found to be photogenerated concomitantly (PB ~50%). These data can be explained by taking into account the shape transition to platelike structures induced in σCS@NSs by aging. It is likely that the diacetylene packing is less effective on flat surfaces, which in turn hinders the monomer-to-polymer conversion. Notwithstanding, if polymerization events occur, blue poly (PCDA) is predominantly formed.

**Figure 5:**
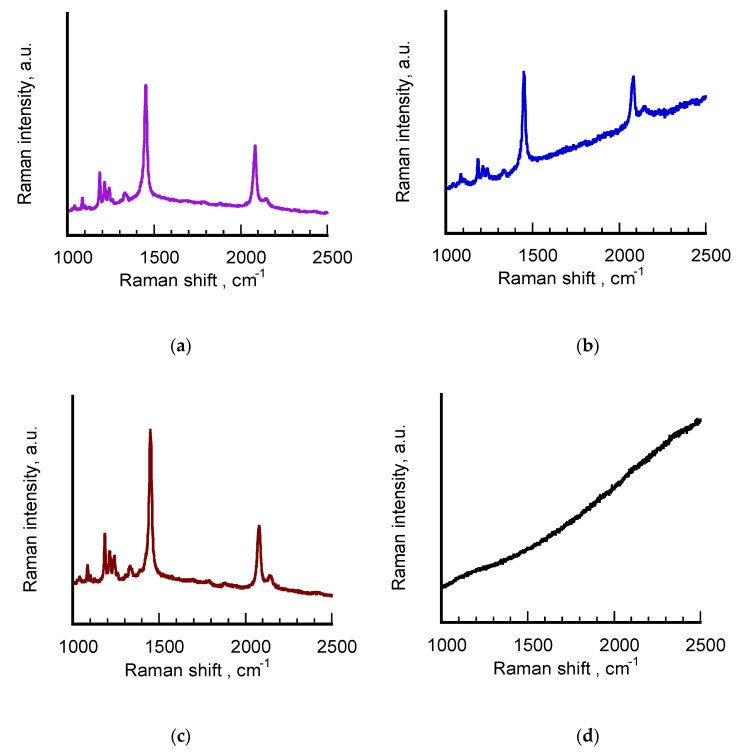
From top to bottom: Raman spectra of hydrosols of freshly-synthesized σ-pPCDA@NSs (purple line) (**a**), aged σ-pPCDA@NSs (blue line) (**b**), aged H-pPCDA@NSs (brown line) (**c**) and pPCDA (black line) (**d**). The purple spectrum was acquired a year after the polymerized sample was prepared. Laser excitation = 632 nm, power beam 1 ÷ 10%.

In order to better investigate the conjugated nature of poly (PCDA) backbone, the polymerized samples were also studied by means of MicroRaman technique in the form of hydrosols. The spectra acquired from aged σ-pPCDA@NSs and H-pPCDA@NSs are reported in Figure 5 together with those of the reference sample pPCDA and the freshly-synthesized σ-pPCDA@NSs recorded a year after preparation. The spectral profiles are limited to 1000–2500 cm^−1^ interval to highlight the presence of the signals corresponding to the double and triple CC bond stretching modes, typical of the conjugated skeleton of polydiacetylenes. Signals assignment to characteristic vibrations according to our previous work [27,29,38] are collected in Table 3.

The Raman spectra of freshly-synthesized σ-pPCDA@NSs (purple line, a) and aged H-pPCDA@NSs (brown line, c) are characterized by the strong peaks at around 2080 and 1450 cm^−1^, typical of the triple and double CC bond stretching modes, respectively, of blue polydiacetylenes. The spectra exhibit the same well-defined, sharp profiles with oscillations in wavenumber values within the instrumental error. In both cases, the higher wavenumber signal is accompanied by a weak peak at around 2150 cm^−1^, attributable to the triple CC bond stretching modes of red poly (PCDA). The corresponding signatures of the double CC bond of the red form above 1500 cm^−1^ are instead not visible, more likely because it is covered by the peaks centered around 1450 cm^−1^, which were acquired in resonant conditions with the excitation line. This result is partially consistent with the UV-vis-NIR spectrum of the sample reported in Figure 4 and the values of Table 2, where the concomitant presence of the red and blue forms in the polymeric shell of the nanostructures was revealed only for freshly-synthesized σ-pPCDA@NSs. Particularly interesting turned out to be the Raman features corresponding to aged σ-pPCDA@NSs (blue line, b). Although the spectral lineshape is dominated by a growing background, corresponding to the fluorescence emission generated by exciting in resonant conditions at 633 nm laser line, the strong and sharp signatures corresponding to blue poly (PCDA) are clearly visible at 2083 and 1499 cm^−1^, respectively, in spite of the very low monomer-to-polymer conversion found for this sample. Also in this case, the weaker peak associated to the polymer in the red phase is present at 2148 cm^−1^. This result can be justified by taking into account the intrinsic SERS properties of these nanostructures induced by their anisotropic geometry. This hypothesis is confirmed by the Raman profile of pPCDA hydrosol (black line) in which the signals corresponding to the polymer present in a comparable amount cannot be detected.

Resuming the results obtained from the study of PCDA-coated nanostructures, the following observations can be made:

- the nanocages of the HCS@NSs sample seem to represent an ideal substrate for the optimum packing of PCDA monomers and their subsequent photopolymerization in the “pure” blue form, suitable for sensing purposes. No significant changes are found in the behavior of the aged nanocages studied in this work with respect to that of the freshly-synthetized ones previously published [21], to indicate that the surface properties of these nanostructures are not altered by aging;
- the nanoplatelets of the aged σCS@NSs sample are found to be platforms much less effective for the chemisorption of PCDA, most probably owing to their flat morphology. By taking into account the well-known selectivity of surfactants for different crystallographic planes of gold nanocrystals in the self-assembly process, it is possible that poor alignment of the carboxylic-endowed diacetylene occurred on the exposed facets of the nanoplatelets, which in turn hampered the polymer photogeneration. Nevertheless, the nanoplatelets turned out to be very performing SERS enhancers in virtue of their well-defined anisotropic shape.
- anchoring and polymerization of PCDA on freshly-synthesized σCS@NSs preserved the morphology of the nanostructures in water, most likely because the formation of a cross-linked structure in the coating shell hindered the shape transition of the metal cores to platelike geometries.

## 4. Conclusions

The use of two chitosans of different molecular mass and acetylation degree (σCS and HCS) allowed the quantitative synthesis of two distinct anisotropic nanostructures, named σCS@NSs and HCS@NSs, from isotropic silver nanoparticles through the same seeded-mediated growth approach. A year after their preparation, a morphological investigation of the two samples revealed that σCS@NSs are composed of gold nanoplatelets with low size dispersion, whereas HCS@NSs are made of highly porous nanocages in Au–Ag alloy. As expected, the chitosan-stabilized nanostructures exhibit plasmonic properties strictly related to their different anisotropic shape, suitable for various application fields and even tunable for targeted purposes.

The versatility of the nanostructures towards post-synthetic reactions was tested by investigating the self-assembly and consequent photopolymerization of PCDA, a carboxyl-terminated diacetylene chosen as a model molecule for the anchoring on noble metal substrates. Different results were obtained from σCS@NSs and HCS@NSs owing to their different, shape-dependent surface properties. In detail, HCS- functionalized nanocages were proved to be optimal substrates for the chemisorption of PCDA molecules, which suggests their profitable use for the design of ad hoc-labeled sensing platforms. Conversely, σCS-stabilized nanoplatelets, although less performing in surface assembly processes, turned out to be highly effective SERS enhancers and could be excellent candidates for the development of diagnostic and theranostic systems thanks to their intense, well-defined absorptions in the NIR window.

## Figures and Tables

**Table 1:** Morphological parameters extracted from FESEM images of Figure 2.

Sample	Size (nm)	Thickness (nm)	Aspect Ratio
σCS@NSs	23 ± 8	7 ± 2	3.3
HCS@NSs	55 ± 10	-	1.2

**Table 2:** Spectroscopic parameters extracted from UV–vis–NIR spectra of Figure 4.

Sample	Aging (Year)	PB (%)	PD 10^5^ (L/mol)
σ-pPCDA@NSs	0	66	5.0
σ-pPCDA@NSs	1	61	0.4
H-pPCDA@NSs	1	73	8.4
pPCDA	0	47	0.8
pPCDA@AgNPs ^1^	0	74	7.8

^1^ Data referring to Ref. [27].

**Table 3:** Signals assignment from Raman spectra of Figure 5.

Sample	ν (C = C) (cm^−1^)	ν (C ≡ C) (cm^−1^)	p(PCDA) Form
σ-pPCDA@NSs (purple line)	1452	2082 2145	blue red
σ-pPCDA@NS (black line)	1449	2083 2148	blue red
H-pPCDA@NSs (brown line)	1499	2079 2144	blue red
pPCDA (black line)-	-	-	-

## Supplementary Materials

The following are available online at https://www.mdpi.com/2079-4991/10/2/224/s1, Figure S1: (a) Photographs of hydrosols of σCS@NSs and HCS@NSs; (b) FTIR-ATR spectra of dried σCS@NSs and lyophilized σCS; (c) FTIR-ATR spectra of dried HCS@NSs and lyophilized HCS, Figure S2: Native FESEM images of σCS@NSs and HCS@NSs and corresponding size distribution histograms, Figure S3: EDS spectra supported by FESEM technique of σCS@NSs and HCS@NSs, Table S1: composition of σCS@NSs and HCS@NSs from Atomic Absorption spectroscopy investigation, Table S2: explanation of names given to the samples.
