# Supplementary material for: Chitosan-Stabilized Noble Metal Nanoparticles: Study of their Shape Evolution and Post-Functionalization Properties
Source: Nanomaterials (Basel). 2020 Jan 28;10(2):224. doi: 10.3390/nano10020224 (PMC7074869; doi:10.3390/nano10020224)
Supplement: Supplementary file 1 [file nanomaterials-10-00224-s001.pdf]

SUPPORTING MATERIALS

Chitosan-stabilized noble metal nanoparticles: study of their shape evolution and post-functionalization properties

Massimo Ottonelli[1], Stefania Zappia[1,2], Anna Demartini[1] and Alloisio Marina[1,*]

[1] Dipartimento di Chimica e Chimica Industriale, Università di Genova, Via Dodecaneso 31 -16146 Genova (Italy); massimo.ottonelli@unige.it, adema.chimica@gmail.com, marina.alloisio@unige.it

[2] *Present address:* Istituto di Scienze e Tecnologie Chimiche "Giulio Natta" (SCITEC), Consiglio Nazionale delle Ricerche (CNR), via Alfonso Corti 12, 20133 Milano (Italy); stefania.zappia@ismac.cnr.it

* Correspondence: marina.alloisio@unige.it; Tel.: +39-010-353-6133

Received: date; Accepted: date; Published: date

Abstract: Noble metal anisotropic nanostructures have achieved a growing interest in both academic and industrial domains mostly because of their shape-dependent plasmonic properties in the near-infrared region. In this paper, gold and gold-silver anisotropic nanostructures were synthetized in very high shape-yields through a wet, seed-mediated approach based on the use of nearly spherical silver nanoparticles as seeds and chitosan as stabilizing agent. Two chitosans of different origin and molecular properties were selected for the synthetic pathway, leading to the formation of variously sized and shaped end products. In detail, quite homogeneous nanoplatelets of about 25-nm size and 7-nm thickness or nearly spherical, highly porous nanocages of about 50-nm size were obtained, depending on the type of polysaccharide employed. The shape transition towards anisotropic morphologies occurred through a slow, spontaneous process, in which the chitosan nature seemed to play a key role. As expected, both nanoplatelets and nanocages exhibit shape-dependent plasmonic features and surface properties tunable for a variety of application fields. To prove this point, the nanostructures were successfully post-functionalized with poly(10,12-pentacosadiynoic acid) (PCDA), a carboxylic-endowed diacetylene able to anchor on noble metal substrates, to obtain versatile, chromic platforms suitable for sensing and spectroscopic purposes.

Keywords: anisotropic noble metal nanoparticles; plasmonic properties; chitosan; galvanic replacement reactions; spontaneous reshaping; diacetylenes

---

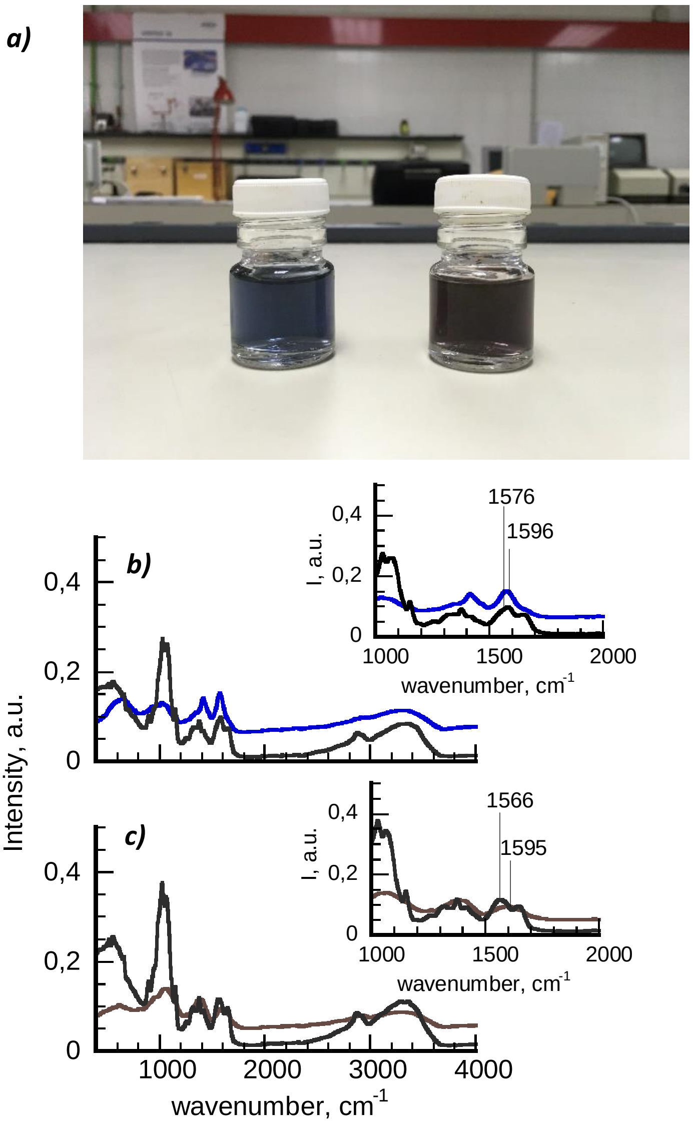

Figure S1. a) Images of hydrosols of σCS@NSs (left) and HCS@NSs (right); **b)** FTIR-ATR spectra of σCS@NSs, dried under vacuum at 25 °C for 48 h, (blue line) and lyophilized powders of σCS (black line), the inset highlights the spectra profiles in the 1000 ÷ 2000 cm^{-1} interval; **c)** FTIR-ATR spectra of HCS@NSs, dried under vacuum at 25 °C for 48 h, (brown line) and lyophilized powders of HCS (black line), the inset highlights the spectra profiles in the 1000 ÷ 2000 cm^{-1} interval.

The spectra of both σCS@NSs and HCS@NSs appear less detailed with respect to that of their corresponding chitosan in form of lyophilized powders, most likely due to residual moisture remaining in the dried samples. Comparing the spectral profiles of pure chitosan with those of chitosan embedded with noble metal nanoparticles, little differences are found for the main signatures with oscillations in the wavenumber values most often within the instrumental error. The one exception is observed for the signal around 1570 cm^{-1}, corresponding to the amide (II)+ NH_2 bending mode,[a] which is about 20-cm^{-1} or 30-cm^{-1} upshifted for σCS@NSs and HCS@NSs, respectively. Based on the current literature, this spectral upshift is associated to the presence of gold nanoparticles linked to chitosan chains.[a] Consequently, FTIR-ATR investigation of our samples provided an indirect confirmation of the AuNSs anchoring to the polysaccharide matrix. Unfortunately, this technique

does not give further information on the type of interactions established between chitosan and the noble metal nanoparticles.

a) Zille A, Fernandes MM, Francesko A, Tzanov T, Fernandes M, Oliveira FR, Almeida L, 471 Amorim T, Carneiro N, Esteves MF, Souto AP. Size and aging effects on antimicrobial efficiency of 472 silver nanoparticles coated on polyamide fabrics activated by atmospheric DBD plasma. ACS Appl 473 Mater Interfaces. 2015;7:13731-44.

a)

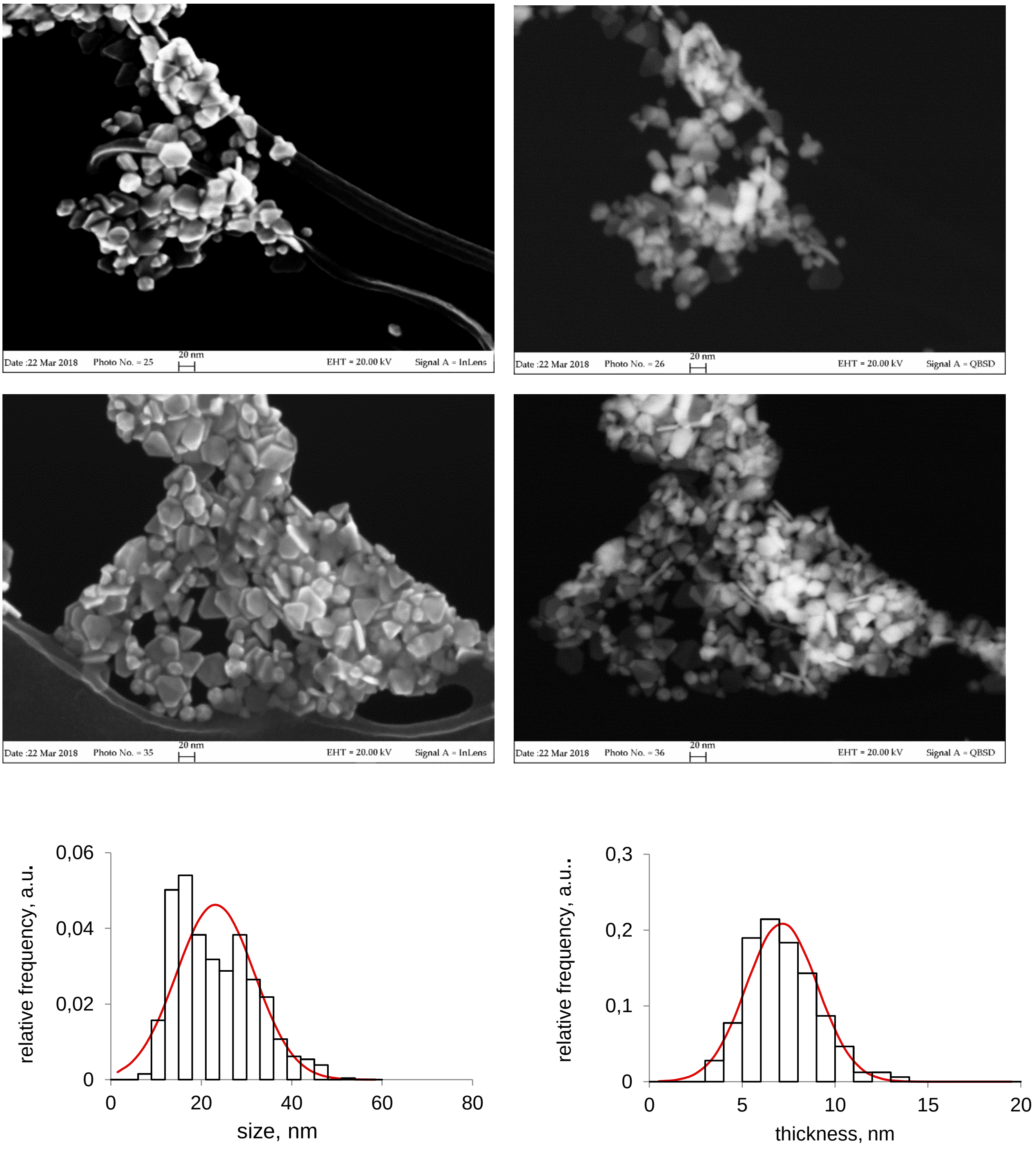

size = 23.1 ± 8.6 (37%)

thickness = 7.1 ± 1.9 (27%)

Figure S2a. Native FESEM images of σCS@NSs acquired in direct (InLens mode, left) and back-scattered (QSBD mode, right) configurations and corresponding size histograms derived from their statistical analysis.

b)

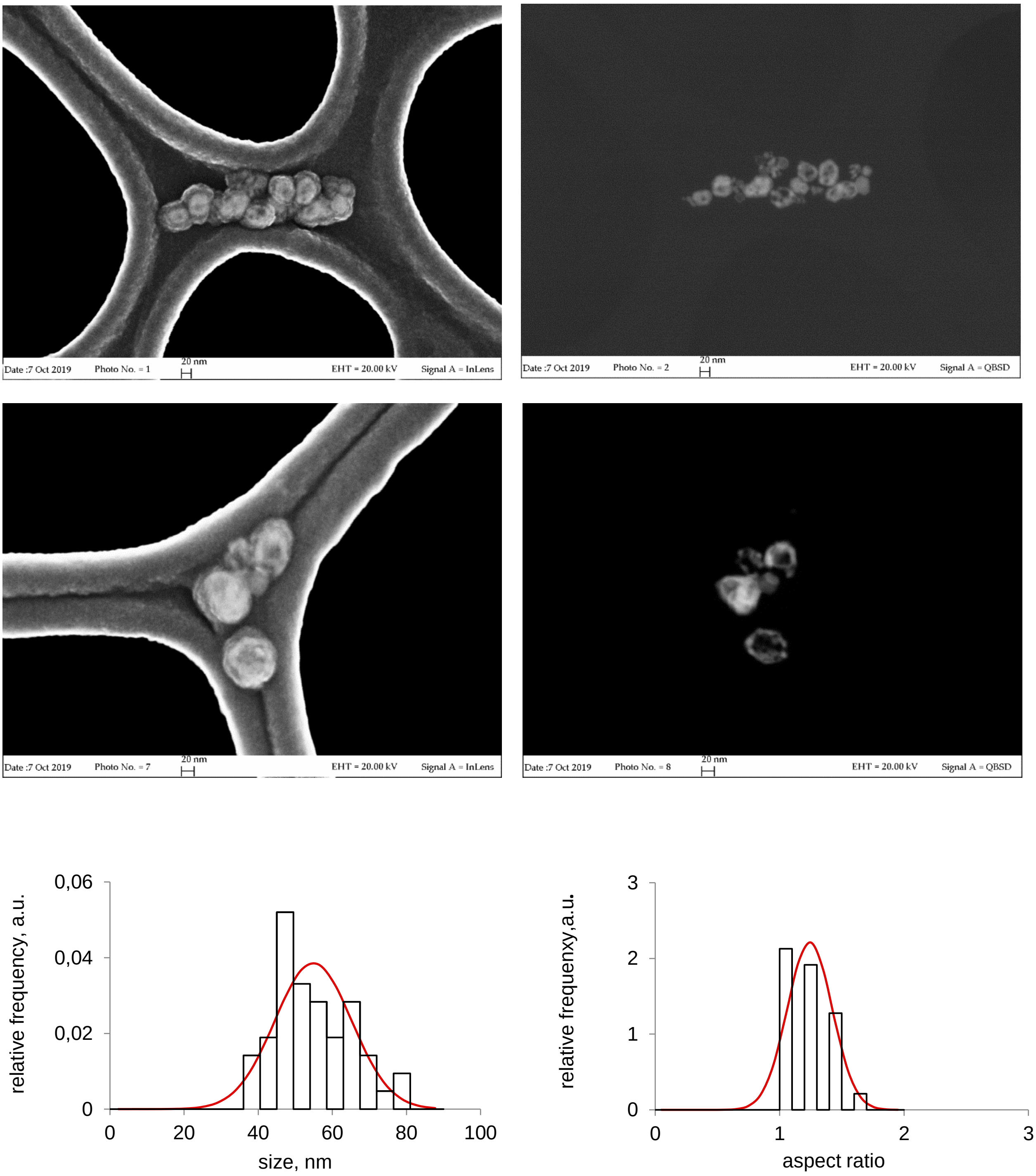

size = 54.8 ± 10.3 (19%)

aspect ratio = 1.2 ± 0.2 (17%)

Figure S2b. Native FESEM images of HCS@NSs acquired in direct (InLens mode, left) and back-scattered (QSBD mode, right) configurations and corresponding size histograms derived from their statistical analysis.

a)

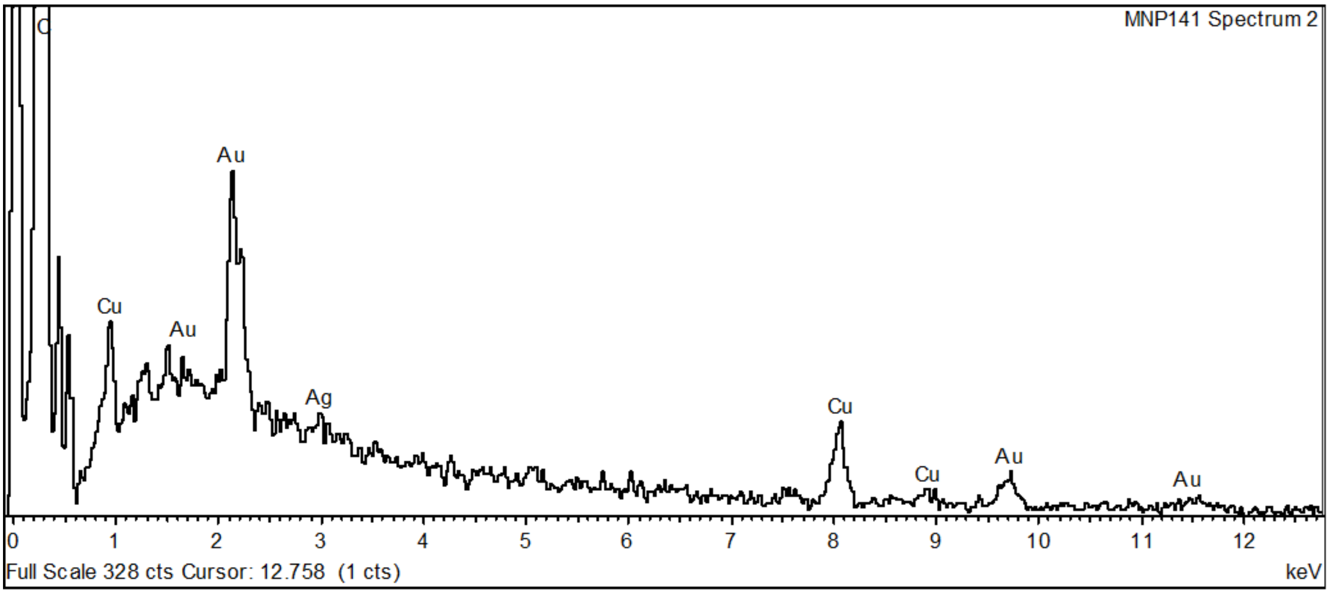

b)

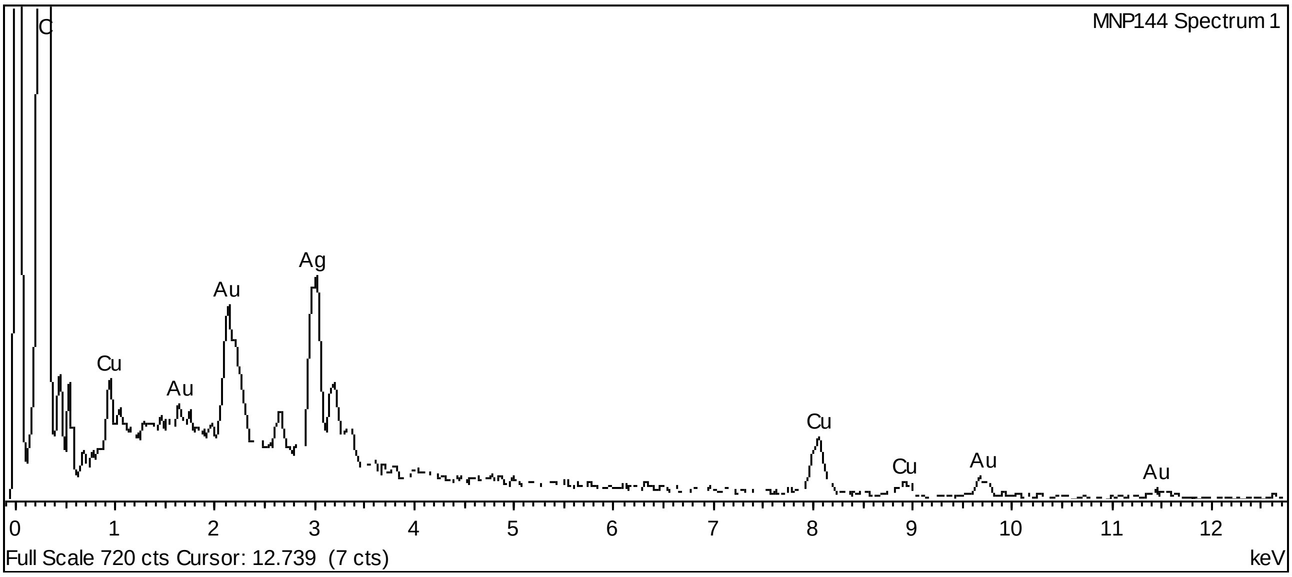

Figure S3. Energy Dispersive X-Ray Spectroscopy (EDS) spectra, supplied by the FESEM technique, of σCS@NSs (**a**) and HCS@NSs (**b**); before the morphological characterization, the samples were sputter-coated with carbon to obtain good conductivity.

Table S1. Data obtained from Atomic Absorption (AA) analysis obtained from diluted hydrosols of σCS@NSs and HCS@NSs.

sample	element	dilution (v/v)	experimental concentration (ppm)	nominal concentration (ppm)
σCS@NSs	Au	1:55	0.8	17.9
σCS@NSs	Ag	1:55	0	89.5
HCS@NSs	Au	1:62	0.3	16.4
HCS@NSs	Ag	1:62	0.1	82.1

Table S2. Explanation of the names given to the samples.

**Sample label**	**Sample Description**
σCS@NSs	nanostructures stabilized with σCS
HCS@NSs	nanostructures stabilized with HCS
σCS@AgNPs	silver nanoparticles stabilized with σCS
HCS@AgNPs	silver nanoparticles stabilized with hCS
σ-PCDA@NSs	σCS-stabilized nanostructures after covering with PCDA
σ-pPCDA@NSs	σCS-stabilized nanostructures after covering with PCDA and photopolymerization
H-PCDA@NSs	HCS-stabilized nanostructures after covering with PCDA
H-pPCDA@NSs	HCS-stabilized nanostructures after covering with PCDA and photopolimerization
pPCDA	PCDA photopolymerized in water-
